# Body fat percentage and the outcomes of hip fractures in adults aged 50 years and above: a 1-year follow-up study

**DOI:** 10.3389/fmed.2025.1588069

**Published:** 2025-06-06

**Authors:** Wenliang Fan, Zhibang Zhao, Liqiang Wang, Qingbo Chu

**Affiliations:** Emergency Trauma Center, Nanyang Second People’s Hospital, Nanyang, Henan, China

**Keywords:** body fat percentage, hip fracture, post-operative outcome, associations, adults aged 50 years or older

## Abstract

**Objective:**

This study aimed to investigate the relationship between body fat percentage (BFP) and postoperative outcomes, including mortality and free ambulation rates, in older adults following hip fracture surgery over a 1-year follow-up period.

**Methods:**

An observational cohort study was conducted at a single trauma center in China from January 2014 to January 2022, enrolling 895 patients (299 males, 596 females) aged ≥50 years with surgically treated hip fractures. BFP was measured via bioimpedance analysis (BIA) at admission. Cox proportional hazards and logistic regression models were employed to assess associations between BFP and outcomes, adjusting for confounders. Restricted cubic splines identified optimal BFP thresholds.

**Results:**

A non-linear relationship between BFP and mortality was observed in both sexes. Optimal BFP ranges were 19.49–27.28% for males and 25.39–32.64% for females. Deviations from these ranges significantly increased mortality risk (adjusted HR for high vs. middle BFP: males 2.27, 95% CI 1.16–4.43; females 2.00, 95% CI 1.15–3.46) and reduced free ambulation rates (*p* < 0.05). Sex-specific differences emerged: high BFP independently predicted poorer outcomes in males, while both low and high BFP were detrimental in females.

**Conclusion:**

Extremes in BFP—either low or high—are associated with elevated mortality and impaired functional recovery after hip fracture surgery, underscoring the dual role of adiposity in postoperative prognosis.

## Background

Hip fractures are a significant cause of morbidity and mortality in older adults, with the global incidence of osteoporotic fractures, particularly hip fractures, projected to increase dramatically (1). The aging population is particularly vulnerable to hip fractures, which often lead to long-term disability, reduced quality of life, and increased healthcare costs (2). Previous studies have identified numerous risk factors associated with hip fractures, including advanced age, low bone mineral density, history of falls, and comorbidities such as osteoporosis and arthritis (3, 4). Understanding the factors that influence the outcomes of hip fractures is crucial for developing effective prevention and treatment strategies.

Body fat is an important component of body composition that has been shown to influence bone health and fracture risk (5). The relationship between body fat and bone health is complex and multifaceted, involving both mechanical and metabolic factors. Body fat can increase mechanical loading on the skeleton, particularly in weight-bearing bones such as the hips and spine, which can stimulate bone formation and increase bone mineral density (6). Additionally, adipose tissue is a significant source of estrogen, particularly in postmenopausal women, which can have a protective effect on bone density (7). Conversely, low BFP in older adults is often associated with poor bone metabolism, muscle atrophy, and reduced bone density, which can compromise recovery from hip fractures (8). Furthermore, low BFP may indicate underlying nutritional deficiencies or systemic illnesses that further weaken bone structure and delay healing processes (9).

However, high BFP is also associated with an increased risk of comorbidities such as diabetes, cardiovascular disease, and metabolic syndrome, which can negatively impact the outcomes of hip fracture surgery (10). Obesity, as indicated by high BFP, can lead to increased surgical site infections, venous thromboembolism, and other postoperative complications, thereby worsening the prognosis of hip fracture patients (11). Additionally, high BFP can impair bone regeneration and healing following fractures due to the negative effects of inflammation and oxidative stress on osteoblast function and bone matrix formation (12). Additionally, high BFP can lead to a pro-inflammatory environment characterized by elevated levels of cytokines such as TNF-*α* and IL-6, which disrupt normal bone remodeling and contribute to osteoporosis (13, 14). The presence of these inflammatory markers not only affects bone health but also increases the risk of surgical complications and reduces the overall success rate of hip fracture surgery (15). High BFP also increases the risk of thromboembolism by promoting a hypercoagulable state and impairing venous return, which is particularly dangerous in post-surgical patients who are already at an elevated risk of developing blood clots (16, 17).

Given the complex and multifaceted relationship between BFP and hip fracture outcomes, it is essential to conduct well-designed studies on the relationship between BFP and the prognosis of hip fracture patients. This study aims to investigate the relationship between BFP and the outcomes of hip fractures in older adults over a 1-year follow-up period, with a particular focus on postoperative mortality and free ambulation rates. To capture the nature of this relationship, we employed Restricted Cubic Splines (RCS), a statistical tool that allows for flexible modeling of multiple types of associations by dividing the range of a variable into segments and fitting a smooth curve across these segments (18). By understanding the role of BFP in hip fracture outcomes, we can develop more effective strategies for the prevention and management of hip fractures in older adults.

## Methods

### Study design

**Figure 1:**
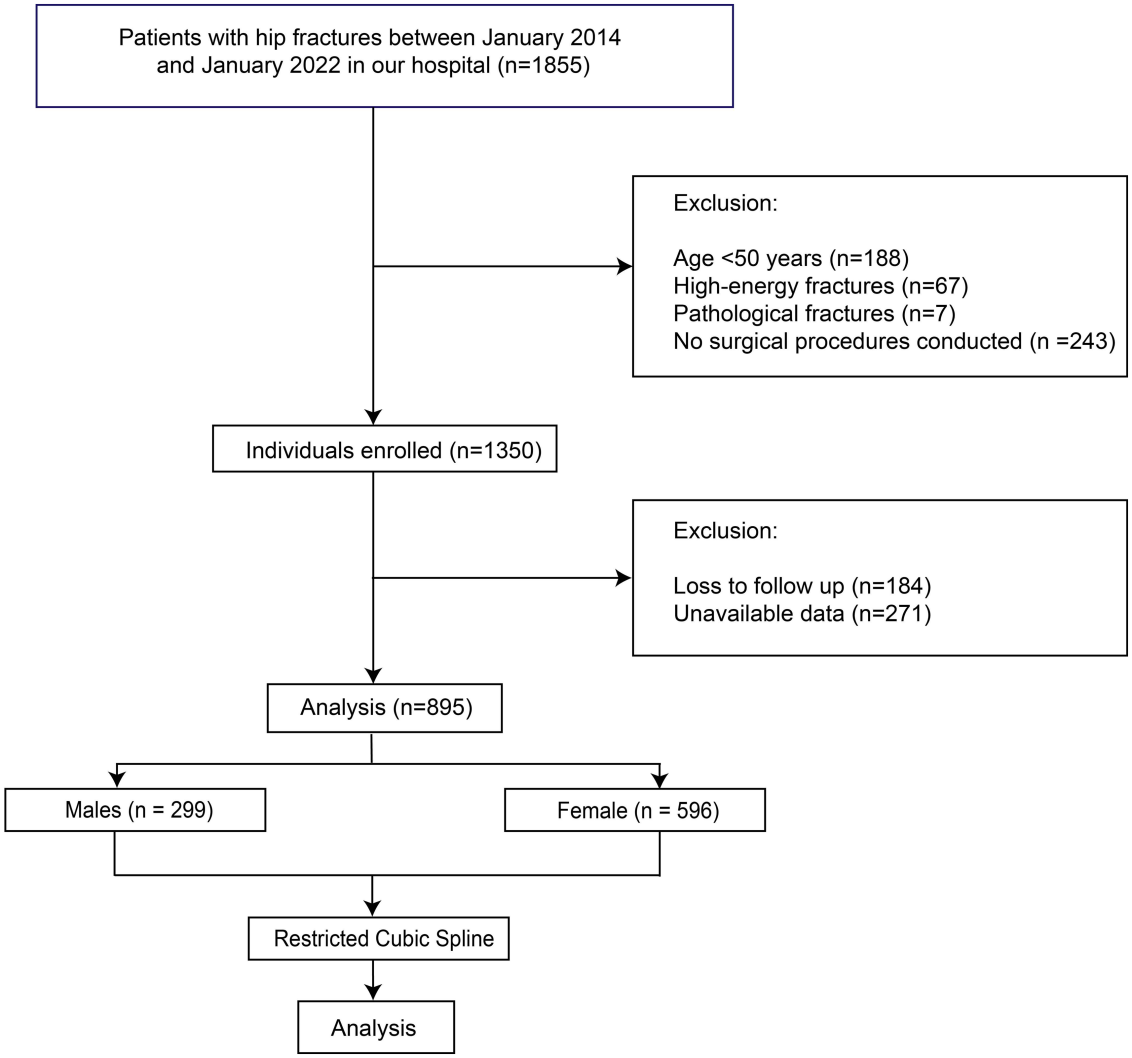
Flow chart of our study.

This research comprised an observational investigation carried out at the Emergency Trauma Center of Nanyang Second People’s Hospital, Nanyang, Henan Province, China. The study was conducted in accordance with the tenets delineated in the Declaration of Helsinki and obtained approval from the Ethics Committee of Nanyang Second People’s Hospital (ID: 2013 Research Review No. 21). Stringent measures were implemented to safeguard patient confidentiality and explicit written consent was procured from all individuals enrolled in the study. The patients who were admitted to our department due to hip fractures (femoral neck fractures or intertrochanteric fractures) between January 2014 and January 2022 and met the inclusion and exclusion criteria were preliminarily enrolled in this study. The inclusion criteria were: A. with femoral neck fractures or intertrochanteric fractures; B. treated with surgery; C. with available and adequate data. The exclusion criteria were: A. younger than 50 years old; B. with pathological fractures; C. with high-energy fractures. The patients who were lost to follow-up were also excluded (Figure 1). To avoid the bias caused by the BFP of different sex, all the analyses in this study were performed by males and females, separately.

### Data collection

Data were retrieved from electronic medical records and included demographic characteristics (age, sex, height, weight), fracture-related features (fracture location, type, history), surgical details (procedures, anesthesia type, time from injury to surgery), comorbidities (hypertension, diabetes, chronic obstructive pulmonary disease, etc.), and laboratory findings (hemoglobin, albumin, glucose, and so on). Charlson comorbidity index (CCI) was employed to assess the presence and impact of these comorbid conditions (19). Electrocardiogram and chest radiograph results were classified as abnormal if they had an impact on hip fracture outcomes. Assessments were independently conducted by two senior physicians, with discrepancies resolved by another senior physician. BFP was measured by using bioimpedance analysis (BIA) based on InBody BWA2.0 (InBody Co. Ltd. China, Shanghai) when patients were admitted to our department.

### Outcomes

The primary outcomes were all-cause mortality at 3 months, 6 months, and 1 year after surgery. Secondary outcomes included free walking abilities at 3 months, 6 months, and 1 year. Free walking ability was defined as the ability to perform activities of daily living without assistance. The enrolled participants were followed up via telephone once a month to collect their survival data. All patients were followed up for 1 year from the date of admission, with no further follow-up conducted beyond this period. Mortality was first ascertained through telephone and confirmed after a review of death records.

### Statistical analysis

Continuous variables were presented as mean ± standard deviation (SD), and categorical variables were expressed as counts (percentages). The CCI was transformed into a binary variable using a cutoff of 4. Normally distributed variables were compared using independent Student’s t-tests, while non-normally distributed variables were analyzed using Wilcoxon rank-sum tests. Categorical data were compared using chi-square tests or Fisher’s exact tests.

Cox proportional hazards models were used to explore the association between BFP and mortality, adjusting for potential confounders. Restricted cubic splines with 4 knots were employed to assess the nonlinear relationships between BFP and outcomes (20), and optimal cutoff points were determined based on hazard ratios (HRs) and confidence intervals (CIs). Patients were divided into groups according to the cutoff points and analyzed using Kaplan–Meier survival analysis and log-rank tests. Multivariate logistic regression analysis was conducted to evaluate the impact of BFP on mortality and free walking ability. All statistical analyses were performed using R software version 4.1.1. A *p*-value <0.05 was considered statistically significant, and for multiple tests, the Bonferroni method was used to adjust the *p*-values.

## Results

### General information

A total of 895 patients were finally included in the analysis (Figure 1), with 299 males and 596 females. The mean age of males was 72.44 ± 10.33 years, and that of females was 72.49 ± 10.51 years. Of all patients, 160 males (53.51%) and 253 females (42.45%) underwent hip arthroplasty, with the remaining patients treated with internal fixation. The overall 1-year mortality rate was 21.07% in males and 16.61% in females. The baseline characteristics of the patients are summarized in Table 1. The mean BFP of males was 24.83% ± 7.19%, and in females it was 29.94% ± 7.73%. In the comparison between the patients of survival > 1 year and survival ≤ 1 year, no significant differences in BFP were identified in both males and females. Therefore, we inferred the existence of a nonlinear relationship between BFP and outcomes.

**Table 1:** Demographic characteristics of males and females with hip fractures stratified by survival status (survival > 1 year vs. survival ≤ 1 year).

Variables	Males (*n* = 299)				Females (*n* = 596)			
	Overall	Survival > 1 year	Survival ≤ 1 year	*p* value	Overall	Survival > 1 year	Survival ≤ 1 year	*p* value
	(*n* = 299)	(*n* = 236)	(*n* = 63)		(*n* = 596)	(*n* = 497)	(*n* = 99)	
Age(years)	72.44 ± 10.33	70.78 ± 10.03	78.65 ± 9.03	<0.001	72.49 ± 10.51	71.15 ± 10.44	79.25 ± 8.02	<0.001
BMI (kg/m2)	21.97 ± 4.20	21.97 ± 4.22	21.99 ± 4.16	0.966	21.69 ± 4.23	21.75 ± 4.21	21.44 ± 4.34	0.444
BFP (%)	24.83 ± 7.19	24.48 ± 5.90	26.13 ± 10.69	0.111	29.94 ± 7.73	29.98 ± 5.75	29.75 ± 14.00	0.536
Fractures history (yes)	40 (13.38%)	30 (12.71%)	10 (15.87%)	0.513	88 (14.77%)	73 (14.69%)	15 (15.15%)	0.906
Smoking history (yes)	33 (11.04%)	25 (10.59%)	8 (12.70%)	0.636	72 (12.08%)	61 (12.27%)	11 (11.11%)	0.746
Alcoholism history(yes)	14 (4.68%)	11 (4.66%)	3 (4.76%)	1.000	36 (6.04%)	28 (5.63%)	8 (8.08%)	0.351
Location of fracture(femoral neck)	176 (58.86%)	136 (57.63%)	40 (63.49%)	0.401	281 (47.15%)	236 (47.48%)	45 (45.45%)	0.712
Surgical procedures(arthroplasty)	160 (53.51%)	124 (52.54%)	36 (57.14%)	0.515	253 (42.45%)	216 (43.46%)	37 (37.37%)	0.263
Anesthesia (spinal)	3 (1.00%)	2 (0.85%)	1 (1.59%)	0.510	6 (1.01%)	6 (1.21%)	0 (0.00%)	0.596
CCI score (>4)	70 (23.41%)	53 (22.46%)	17 (26.98%)	0.451	153 (25.67%)	126 (25.35%)	27 (27.27%)	0.690
Electrocardiogram (abnormal)	169 (56.52%)	139 (58.90%)	30 (47.62%)	0.109	347 (58.22%)	292 (58.75%)	55 (55.56%)	0.556
Chest radiograph (abnormal)	153 (51.17%)	123 (52.12%)	30 (47.62%)	0.526	302 (50.67%)	252 (50.70%)	50 (50.51%)	0.971
Hypertension(yes)	160 (53.51%)	129 (54.66%)	31 (49.21%)	0.441	354 (59.40%)	306 (61.57%)	48 (48.48%)	0.015
Polytrauma(yes)	43 (14.38%)	33 (13.98%)	10 (15.87%)	0.704	87 (14.60%)	67 (13.48%)	20 (20.20%)	0.084
Time from injury to surgery (Days)	4.83 ± 0.94	4.81 ± 0.95	4.94 ± 0.90	0.235	4.91 ± 0.93	4.91 ± 0.94	4.93 ± 0.87	0.650
RBC (10^12/L)	4.58 ± 0.57	4.56 ± 0.58	4.63 ± 0.51	0.406	4.54 ± 0.55	4.55 ± 0.55	4.51 ± 0.56	0.487
Hb (g/L)	96.46 ± 13.72	95.97 ± 13.86	98.31 ± 13.11	0.290	95.99 ± 14.26	96.15 ± 14.30	95.15 ± 14.12	0.465
ALB (g/L)	38.58 ± 9.18	38.61 ± 9.16	38.50 ± 9.35	0.944	38.10 ± 8.85	38.24 ± 8.97	37.42 ± 8.23	0.485
GLU (mmol/L)	6.31 ± 1.39	6.35 ± 1.41	6.16 ± 1.31	0.424	6.26 ± 1.40	6.29 ± 1.38	6.11 ± 1.50	0.279

BFP, Body Fat percentage; BMI, Body Mass Index; CCI, Charlson Comorbidity Index; Hb, Hemoglobin; INR, International Normalized Ratio; GLU, Blood Glucose; ALB, Albumin.

### Nonlinear relationships

**Figure 2:**
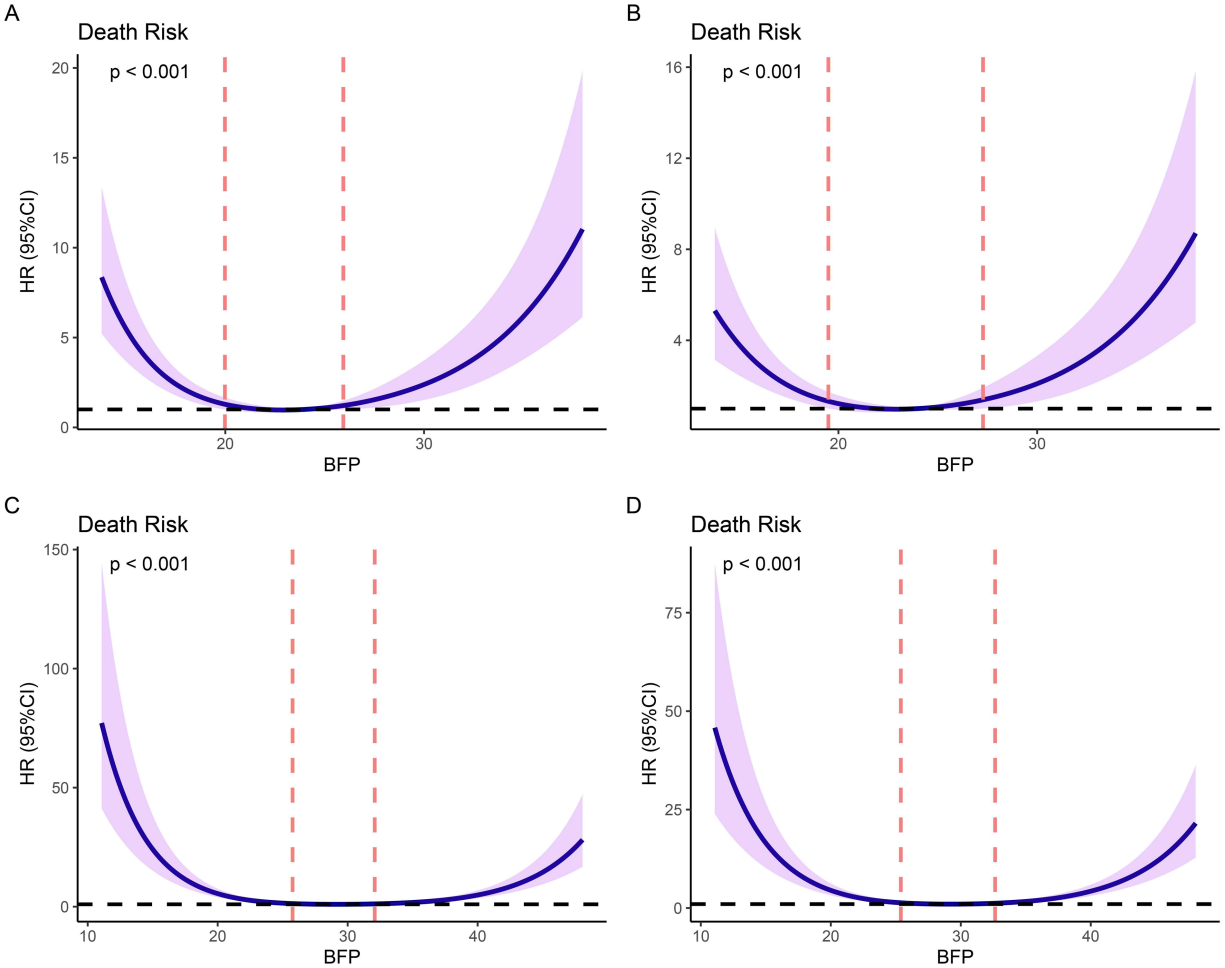
Restricted cubic splines (RCS) based on Cox proportional hazards models. **(A)** non-adjusted models based on males; **(B)** models adjusted for age based on males; **(C)** non-adjusted models based on females; **(D)** models adjusted for age and hypertension based on females.

Based on Cox models, we established the restricted cubic spline models and adjusted the models by baseline variables with significant differences (Figure 2). Restricted cubic spline analysis revealed a U-shaped relationship between BFP and 1-year mortality in both males and females. In males, the lowest death risk was observed at a BFP of 22.90%, with increased risks at both lower and higher BFP levels (Figure 2A). After adjusting for age, the U-shaped relationship remained significant (Figure 2B). In females, the lowest death risk was observed at a BFP of 29.11% (Figure 2C). After adjusting for age and hypertension, the U-shaped relationship was still evident, with higher risks at both lower and higher BFP levels (Figure 2D). According to the curves, the cutoff values were identified (for males: low BFP: ≤ 19.49%; middle BFP: 19.49–27.28%; high BFP: ≥27.28%; for females: low BFP: ≤ 25.39%; middle BFP: 25.39–32.64%; high BFP: ≥32.64%). This U-shaped relationship indicates that there is an optimal BFP range associated with the best post-surgical outcomes. Deviations from this range, either too low or too high, are associated with increased mortality and reduced functional recovery, highlighting the importance of maintaining BFP within the identified optimal range for improving prognosis following hip fracture surgery.

### Outcomes

**Figure 3:**
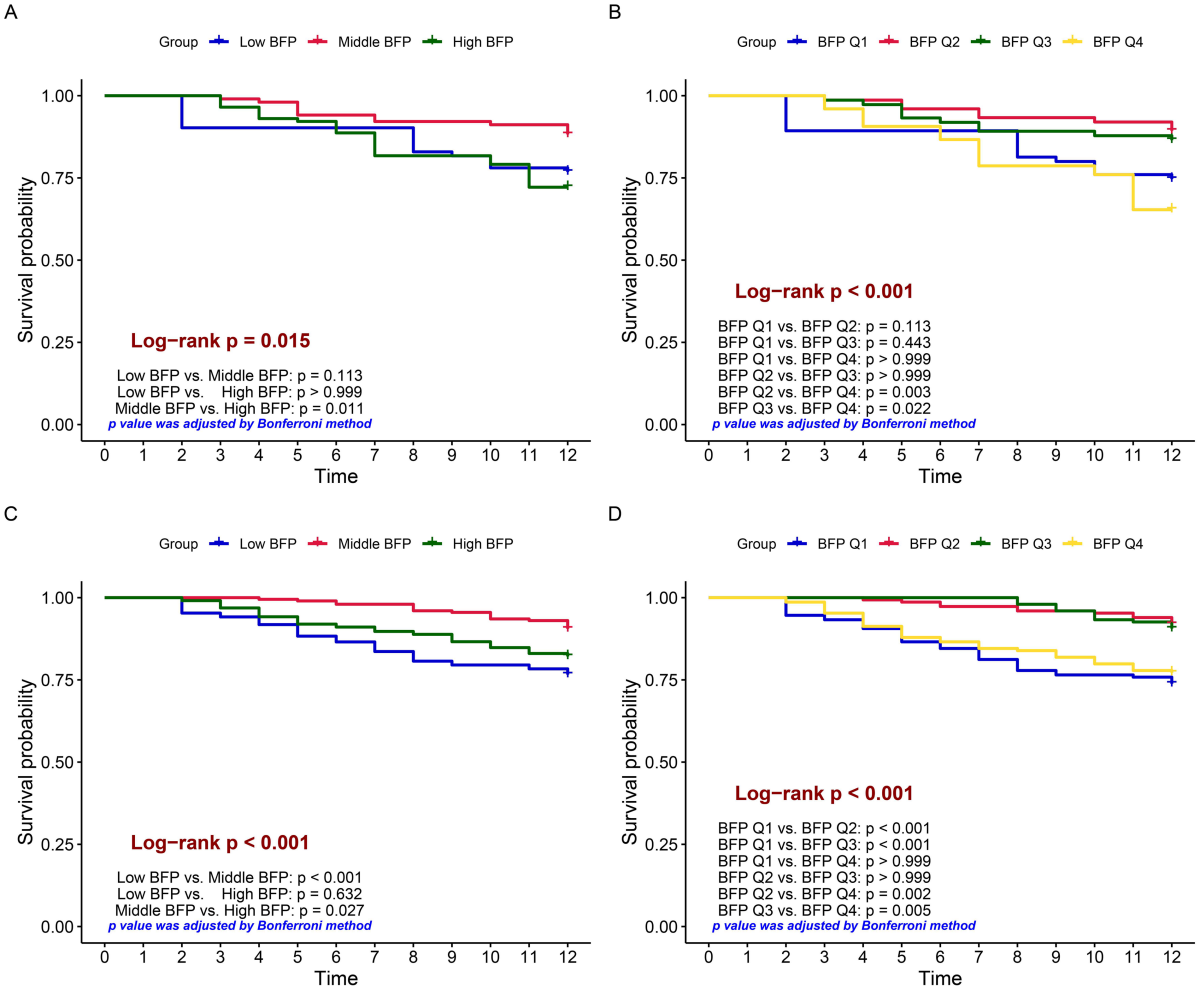
Kaplan–Meier survival curves comparing survival probabilities among different groups. Log-rank tests were used to compare survival differences, and *p*-values were corrected by the Bonferroni method. **(A)** grouped by low, middle, and high BFP based on males; **(B)** grouped by BFP quartiles (Q1 to Q4) based on males; **(C)** grouped by low, middle, and high BFP based on females; **(D)** grouped by BFP quartiles (Q1 to Q4) based on females.

Based on the RCS cutoff values, individuals were categorized into three BFP groups: low BFP, middle BFP, and high BFP. Moreover, they were also divided into quartiles (Q1-Q4). Kaplan–Meier survival analysis showed significant differences in survival rates between different BFP groups. In males, the high BFP group had a lower survival probability compared to the middle BFP group (*p* = 0.011), while no significant difference was found between low BFP and middle BFP (*p* = 0.113) or low BFP and high BFP (*p* > 0.999). When analyzed by quartiles (Figure 3B), significant differences were observed between BFP Q2 vs. BFP Q4 (*p* = 0.003) and BFP Q3 vs. BFP Q4 (*p* = 0.022), indicating that higher BFP was associated with lower survival rates (Figure 3B). In females, the middle BFP group had a higher survival probability compared to the low BFP group (*p* < 0.001) and the high BFP group (*p* = 0.027). When analyzed by quartiles (Figure 3D), significant differences were observed between BFP Q1 vs. BFP Q2 (*p* < 0.001), BFP Q1 vs. BFP Q3 (*p* < 0.001), BFP Q2 vs. BFP Q4 (*p* = 0.002), and BFP Q3 vs. BFP Q4 (*p* = 0.005), indicating that both lower and higher BFP were associated with lower survival rates.

**Figure 4:**
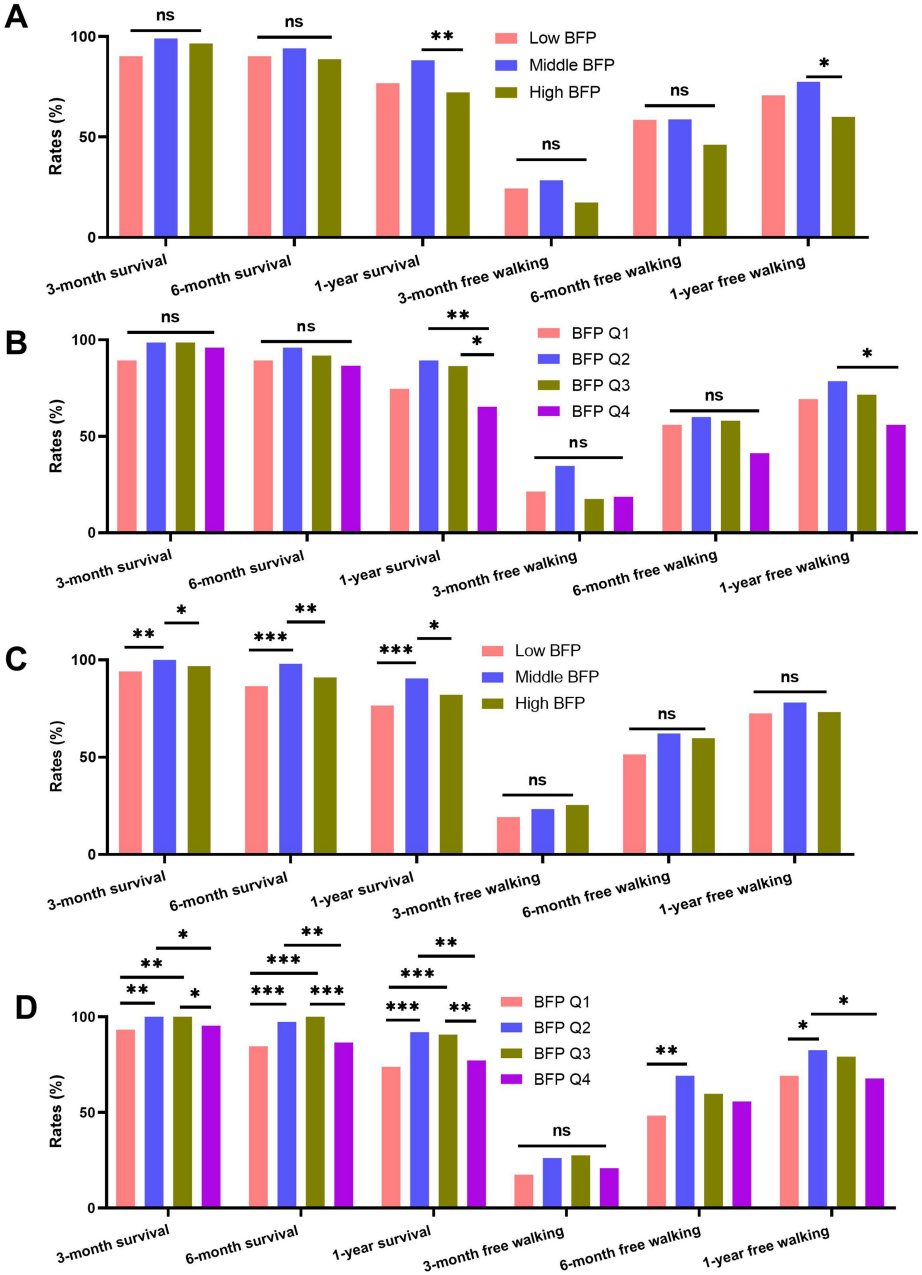
Comparison of survival and free walking ability rates at 3 months, 6 months, and 1 year post-surgery across different BFP groups (low BFP, middle BFP, high BFP) and BFP quartiles (Q1 to Q4). **(A)** males grouped by low, middle, and high BFP; **(B)** males grouped by BFP quartiles; **(C)** males grouped by low, middle, and high BFP; **(D)** males grouped by BFP quartiles.

For males, as shown in Figures 4A,B, there were significant differences in 1-year survival rates and free walking rates across different BFP categories (for 1-year survival rates: high BFP vs. middle BFP, BFP Q2 vs. BFP Q4, BFP Q3 vs. BFP Q4; for 1-year free walking rates: high BFP vs. middle BFP, BFP Q2 vs. BFP Q4, all *p* < 0.05). Similarly, for females (Figures 4C,D), significant differences were observed in survival rates and free walking ability. The females with high BFP and low BFP have significantly lower survival rates at 3 months, 6 months, and 1 year after surgery compared with those with middle BFP (all *p* < 0.05). Moreover, similar differences were also proven in the patients grouped by BFP quartiles (Figure 4D). The baseline variables of patients categorized by different categories were summarized in Supplementary Tables 1, 2.

### Predictive values of BFP

The predictive values of BFP for outcomes were analyzed using Cox proportional hazards models and logistic regression models. The results of univariate Cox and logistic models were summarized in Supplementary Tables 3–5, and the significant factors in univariate models were included in multivariate models.

In the Cox models (Table 2), for both males and females, no significance of continuous BFP was proven. In the multivariate logistic models based on males, high BFP was significantly associated with high mortality risk compared with middle BFP, while low BFP was not significant. Similarly, compared with BFP Q2, only BFP Q4 was proven to be a significant risk factor. For females, both low and high BFP, as well as BFP Q1 and BFP Q4, were estimated to be significant risk factors.

**Table 2:** Cox regression for mortality of BFP.

Variables	Males				Females			
	Univariate model		Multivariate model		Univariate model		Multivariate model	
	HR (95% CI)	*p*	HR (95% CI)	*p*	HR (95% CI)	*p*	HR (95% CI)	*p*
BFP (continuous)	1.029 [0.992, 1.068]	0.123	1.028 [0.993, 1.065]	0.114	0.994 [0.964, 1.025]	0.688	0.998 [0.969, 1.027]	0.886
Low BFP	2.145 [1.041, 4.420]	0.038	1.817 [0.878, 3.759]	0.107	2.793 [1.618, 4.822]	<0.001	2.536 [1.463, 4.395]	0.001
Middle BFP	Ref	Ref	Ref	Ref	Ref	Ref	Ref	Ref
High BFP	2.583 [1.330, 5.016]	0.005	2.271 [1.164, 4.431]	0.016	2.023 [1.172, 3.493]	0.011	1.995 [1.151, 3.459]	0.014
BFP Q1	2.650 [1.160, 6.054]	0.021	2.128 [0.927, 4.887]	0.075	3.762 [1.970, 7.187]	<0.001	3.629 [1.897, 6.942]	<0.001
BFP Q2	Ref	Ref	Ref	Ref	Ref	Ref	Ref	Ref
BFP Q3	1.306 [0.516, 3.310]	0.573	1.131 [0.445, 2.875]	0.796	1.161 [0.537, 2.509]	0.705	1.219 [0.563, 2.640]	0.616
BFP Q4	3.699 [1.674, 8.173]	0.001	3.216 [1.452, 7.125]	0.004	3.172 [1.642, 6.126]	0.001	3.256 [1.682, 6.305]	<0.001

BFP, Body Fat percentage; HR, Hazard Ratio; CI, Confidence Interval. Multivariate models were adjusted for age in males and for age and hypertension in females.

The results of multivariate logistics models were summarized in Table 3. For males, BFP (continuous) was significantly associated with 1-year mortality and 1-year free walking ability. Similar to the results of Cox models based on males, instead of low BFP, high BFP was estimated to relate to a high risk of 1-year mortality and a low risk of 1-year free walking ability compared with middle BFP. These results were similar in quartile BFP. For females, no significance of continuous BFP was detected. Low and high BFP showed a significant trend for 6-month mortality and 1-year mortality. BFP Q1 and Q4 were associated with a significant increase in 6-month and 1-year mortality and with a significant decrease in 6-month and 1-year free walking ability compared with BFP Q2.

**Table 3:** Logistic regression analysis for free walking ability at 6 months and 1-year post-surgery of BFP.

Variables	6-month mortality		1-year mortality		6-month free walking ability		1-year free walking ability	
	OR (95% CI)	*p*	OR (95% CI)	*p*	OR (95% CI)	*p*	OR (95% CI)	*p*
Males								
BFP (continuous)	1.000 [0.950, 1.053]	0.991	1.040 [1.001, 1.082]	0.047	0.970 [0.939, 1.002]	0.068	0.961 [0.928, 0.995]	0.026
Low BFP	1.444 [0.468, 4.659]	0.523	1.851 [0.818, 4.310]	0.143	1.081 [0.593, 1.977]	0.799	0.802 [0.405, 1.589]	0.526
Middle BFP	Ref	Ref	Ref	Ref	Ref	Ref	Ref	Ref
High BFP	1.803 [0.666, 5.401]	0.261	2.780 [1.330, 6.123]	0.008	0.609 [0.352, 1.047]	0.074	0.445 [0.239, 0.812]	0.009
BFP Q1	2.174 [0.582, 10.436]	0.275	2.255 [0.906, 6.025]	0.089	0.944 [0.487, 1.829]	0.863	0.717 [0.333, 1.524]	0.39
BFP Q2	Ref	Ref	Ref	Ref	Ref	Ref	Ref	Ref
BFP Q3	1.864 [0.462, 9.238]	0.398	1.179 [0.421, 3.386]	0.754	0.945 [0.487, 1.831]	0.866	0.706 [0.325, 1.512]	0.372
BFP Q4	2.976 [0.837, 13.979]	0.116	4.389 [1.826, 11.537]	0.001	0.485 [0.249, 0.933]	0.031	0.358 [0.169, 0.734]	0.006
Females								
BFP (continuous)	0.999 [0.963, 1.035]	0.936	1.003 [0.976, 1.031]	0.812	1.007 [0.986, 1.030]	0.502	0.991 [0.967, 1.015]	0.44
Low BFP	6.569 [2.414, 23.046]	0.001	2.646 [1.438, 5.008]	0.002	0.716 [0.465, 1.102]	0.129	0.845 [0.517, 1.381]	0.502
Middle BFP	Ref	Ref	Ref	Ref	Ref	Ref	Ref	Ref
High BFP	4.525 [1.644, 15.970]	0.008	2.081 [1.141, 3.908]	0.019	1.002 [0.667, 1.505]	0.991	0.823 [0.517, 1.303]	0.407
BFP Q1	6.034 [2.195, 21.324]	0.001	3.944 [1.956, 8.452]	<0.001	0.445 [0.271, 0.723]	0.001	0.518 [0.292, 0.904]	0.022
BFP Q2	Ref	Ref	Ref	Ref	Ref	Ref	Ref	Ref
BFP Q3	0.000 [0.000, Inf]	0.985	1.252 [0.543, 2.932]	0.598	0.686 [0.419, 1.120]	0.133	0.817 [0.449, 1.477]	0.504
BFP Q4	5.629 [2.012, 20.099]	0.003	3.668 [1.796, 7.947]	0.001	0.600 [0.367, 0.976]	0.04	0.452 [0.255, 0.787]	0.006

BFP, Body Fat percentage; HR, Hazard Ratio; CI, Confidence Interval. Multivariate models for 6-month mortality were adjusted for age and electrocardiogram in males and for age and hypertension for females; multivariate models for 1-year mortality were adjusted for age in males and for age and hypertension for females; multivariate models for 6-month free walking ability were adjusted for age in males and for age, surgical procedures, location of fracture, and hypertension for females; multivariate models for 1-year free walking ability were adjusted for age in males and for age, surgical procedures, location of fracture, and hypertension for females.

## Discussion

Our study revealed a non-linear relationship between BFP and 1-year mortality in older adults following hip fracture surgery, with optimal BFP ranges identified at 19.49–27.28% for males and 25.39–32.64% for females. Both lower and higher BFP levels were associated with increased mortality risk and reduced free ambulation rates. These findings underscore the dual role of body fat in post-fracture outcomes, where extremes in adiposity—either insufficient or excessive—may independently compromise recovery through distinct mechanisms. This may be due to the fact that low BFP is often linked to malnutrition, which can lead to muscle atrophy and reduced bone density, making it difficult for patients to recover from surgery and increasing the risk of complications (21, 22). On the other hand, high BFP is frequently associated with a higher incidence of comorbidities, such as diabetes, cardiovascular disease, and metabolic syndrome (23, 24). These conditions can negatively impact the outcomes of hip fracture surgery, increase the risk of postoperative complications, and impair bone healing and functional recovery (25). Therefore, maintaining an appropriate BFP level is crucial for improving the prognosis of hip fracture patients.

Notably, sex-specific differences emerged in our analysis. While females exhibited a broader optimal BFP range (25.39–32.64%) compared to males (19.49–27.28%), both sexes shared a nonlinear mortality risk pattern. This divergence may reflect hormonal influences, as adipose-derived estrogen in postmenopausal women could partially mitigate bone loss and inflammation, whereas males with high BFP face heightened metabolic dysregulation (26). Pana et al. conducted a prospective population cohort study involving 14,129 participants from the EPIC-Norfolk study, with a median follow-up of 16.4 years (5). They found that higher BFP was associated with a lower risk of hip fractures in women, while in men, higher BFP (above 23%) was associated with an increased risk of hip fractures. This study highlighted the gender differences in the relationship between BFP and fracture risk, suggesting that the protective effect of higher BFP in women may be attributed to increased bone mineral density (BMD) due to greater mechanical loading and hormonal influences. Moayyeri et al. conducted a prospective population study involving 25,639 participants, examining the relationship between body fat mass and osteoporotic fractures (7). They found that body fat mass was a significant predictor of fracture risk in women but not in men. This study further emphasized the importance of body composition in understanding fracture risk and highlighted the need for more detailed assessments of body fat distribution and its impact on bone health.

The secondary outcome of free walking rates further emphasized the clinical relevance of BFP. High BFP in males and both low and high BFP in females predicted reduced mobility, likely due to sarcopenia-driven frailty or obesity-related functional limitations. This aligns with evidence that excess adiposity impairs physical performance, while sarcopenia diminishes resilience to rehabilitation (27, 28).

The distinct optimal BFP ranges identified for males and females (19.49–27.28% vs. 25.39–32.64%) highlight the influence of biological differences on post-fracture outcomes. Males within this range had a significantly lower BFP compared to females (23.12 ± 1.96 vs. 29.10 ± 2.00, *p* < 0.001). Females, particularly postmenopausal women, benefit from adipose tissue as a source of estrogen, which may help counteract bone loss and reduce inflammation, thereby potentially explaining the broader BFP range associated with better outcomes in this group (29). In contrast, males with higher BFP may experience greater metabolic dysregulation and inflammation, leading to worse surgical outcomes. These findings align with previous research indicating that hormonal factors play a significant role in bone health and fracture risk, emphasizing the need for sex-specific considerations in managing hip fracture patients (30, 31).

Our study advances the current understanding of the relationship between body fat and hip fracture outcomes by providing novel evidence on sex-specific optimal BFP ranges and their differential associations with mortality and functional recovery. These findings offer valuable insights for tailoring postoperative care and developing targeted interventions to improve the prognosis of hip fracture patients based on their BFP status and sex. Nutritional interventions to prevent sarcopenia in low-BFP patients and weight management strategies for high-BFP individuals may optimize recovery. Additionally, rehabilitation programs should address both muscle strengthening and metabolic health, particularly in males with elevated BFP.

This study has several limitations. First, its observational design precludes causal inferences, and residual confounding (e.g., dietary patterns, fat distribution, or muscle quality) may influence outcomes. Second, BFP was measured via BIA, which is less precise than DXA, potentially introducing measurement error. Third, the single-center cohort limits generalizability, and selection bias may exist due to the exclusion of patients lost to follow-up. Finally, the cross-sectional assessment of BFP at admission does not account for dynamic changes in body composition during recovery.

Prospective studies should validate BFP thresholds in diverse populations and explore mechanisms linking adiposity to bone health, such as inflammation, hormonal pathways, and fat-muscle crosstalk. Interventions targeting osteosarcopenia—concurrent osteoporosis and sarcopenia—warrant evaluation, as combined resistance training and nutritional supplementation may synergistically improve musculoskeletal health (32, 33). Additionally, advanced imaging techniques to differentiate visceral and subcutaneous fat could elucidate site-specific risks, as visceral adiposity may uniquely drive metabolic dysfunction (34).

## Conclusion

In conclusion, BFP demonstrates a U-shaped relationship with mortality and functional outcomes following hip fracture, emphasizing the need to avoid extremes in adiposity. Integrating body composition analysis into clinical practice could refine risk stratification and guide targeted interventions, ultimately improving survival and mobility in this vulnerable population.

## Data Availability

The raw data supporting the conclusions of this article will be made available by the authors, without undue reservation.

## References

[1] Liu M Yang C Chu Q Fu X Zhang Y Sun G. Superoxide dismutase and glutathione reductase as indicators of oxidative stress levels may relate to geriatric hip fractures' survival and walking ability: a propensity score matching study. Clin Interv Aging. (2022) 17:1081–90. doi: 10.2147/CIA.S370970, PMID: 35855743 PMC9288178

[2] Hansen CM Gadgaard NR Vandenbroucke-Grauls C Hailer N Pedersen A. Interaction between multimorbidity and hip fracture surgery leads to excess risk of infection: a Danish registry-based cohort study of 92,599 patients with hip fracture. Clin Epidemiol. (2025) 17:167–76. doi: 10.2147/CLEP.S507252, PMID: 40027399 PMC11869750

[3] de Fraiture EJ Nijdam TMP van Eerten FJC Schuijt HJ Bikker A Koenderman L. Exploring the role of systemic inflammation in guiding clinical decision making for geriatric patients with a hip fracture. Eur J Trauma Emerg Surg. (2025) 51:192. doi: 10.1007/s00068-025-02875-x, PMID: 40325151 PMC12053202

[4] Carranza FH Arroba CM Corbatón-Anchuelo A Díaz-Guerra GM Pareja FB. Hip fractures and type 2 diabetes in the elderly: risk factors analysis of the Nedices cohort. Diabetes Metab. (2025):101656. doi: 10.1016/j.diabet.2025.101656, PMID: 40268160

[5] Pana TA Kioh SH Neal SR Tan MP Mat S Moayyeri A. Body fat percentage and the long-term risk of fractures. The EPIC-Norfolk prospective population cohort study. Maturitas. (2023) 168:71–7. doi: 10.1016/j.maturitas.2022.11.005, PMID: 36502648 PMC7614563

[6] Amin H Syed FA Khan MA Sultan Z Bukhari M. Partial body fat percentage as a predictor of fragility fractures in a large cohort: a cross-sectional study. Rheumatol Adv Pract. (2024) 8:rkae010. doi: 10.1093/rap/rkae010, PMID: 38390590 PMC10882437

[7] Moayyeri A Luben RN Wareham NJ Khaw KT. Body fat mass is a predictor of risk of osteoporotic fractures in women but not in men: a prospective population study. J Intern Med. (2012) 271:472–80. doi: 10.1111/j.1365-2796.2011.02443.x, PMID: 21848670

[8] Ge J Sun S Zeng J Jing Y Ma H Qian C. Development and validation of machine learning models for predicting low muscle mass in patients with obesity and diabetes. Lipids Health Dis. (2025) 24:162. doi: 10.1186/s12944-025-02577-8, PMID: 40301848 PMC12039300

[9] Carrilho LAO Juliani FL Moreira RCL Dias L Santos F Padilha D. Adipose tissue characteristics as a new prognosis marker of patients with locally advanced head and neck cancer. Front Nutr. (2025) 12:1472634. doi: 10.3389/fnut.2025.1472634 40161297 PMC11949816

[10] Hou L Wang X Li P Zhang H Yao Y Liu Z. Adiposity modifies the association between heart failure risk and glucose metabolic disorder in older individuals: a community-based prospective cohort study. Cardiovasc Diabetol. (2024) 23:318. doi: 10.1186/s12933-024-02418-5, PMID: 39192249 PMC11350974

[11] Amin RM Raad M Rao SS Musharbash F Best MJ Amanatullah DF. Survival bias may explain the appearance of the obesity paradox in hip fracture patients. Osteoporos Int. (2021) 32:2555–62. doi: 10.1007/s00198-021-06046-7, PMID: 34245343 PMC8819709

[12] Lumeng CN Saltiel AR. Inflammatory links between obesity and metabolic disease. J Clin Invest. (2011) 121:2111–7. doi: 10.1172/JCI57132, PMID: 21633179 PMC3104776

[13] Bianchi F Roccabianca P Vianello E Gentile G la Sala L Bandera F. Inhibition of DPP-4 attenuates Endotoxemia-induced NLRC4 Inflammasome and inflammation in visceral adipose tissue of mice fed a high-fat diet. Biomol Ther. (2025) 15:333. doi: 10.3390/biom15030333, PMID: 40149869 PMC11940500

[14] Bluher M. An overview of obesity-related complications: the epidemiological evidence linking body weight and other markers of obesity to adverse health outcomes. Diabetes Obes Metab. (2025) 27:3–19. doi: 10.1111/dom.16263, PMID: 40069923 PMC12000860

[15] Reynoso-Roa AS Gutierrez-Rubio SA Magallon-Gastelum E. The role of Resistin in macrovascular and microvascular complications of type 2 diabetes. Life (Basel). (2025) 15:585. doi: 10.3390/life15040585, PMID: 40283140 PMC12028410

[16] Markova I Huttl M Gayova N. Visceral adipose tissue inflammation and vascular complications in a rat model with severe dyslipidemia: sex differences and PAI-1 tissue involvement. Biomol Ther. (2024) 15:19. doi: 10.3390/biom15010019, PMID: 39858414 PMC11763299

[17] Littlejohn JB Grenn EE Carter KT Hazlewood R Sitta J Florez E. Adiposity and coagulation: predicting Postinjury coagulation with advanced imaging analysis. J Surg Res. (2023) 292:190–6. doi: 10.1016/j.jss.2023.07.048, PMID: 37633248 PMC10658990

[18] Chen L Gao F Wang X Fan W Shen X Wang J. The U-curve associations of birth interval with prevalence of osteoarthritis in postmenopausal women. Aging Clin Exp Res. (2025) 37:144. doi: 10.1007/s40520-025-03057-w, PMID: 40338470 PMC12062161

[19] Liu M Ji S Yang C Zhang T Han N Pan Y. Prealbumin as a nutrition status indicator may be associated with outcomes of geriatric hip fractures: a propensity score matching and 1-year follow-up study. Aging Clin Exp Res. (2022) 34:3005–15. doi: 10.1007/s40520-022-02243-4, PMID: 36127624

[20] Liu M Chu Q Yang C Wang J Fu M Zhang Z. The paradoxical relation between serum uric acid and outcomes of hip fracture in older patients after surgery: a 1-year follow-up study. Surgery. (2022) 172:1576–83. doi: 10.1016/j.surg.2022.07.008, PMID: 36031447

[21] Garber AK Bennett JP Wong MC Tian IY Maskarinec G Kennedy SF. Cross-sectional assessment of body composition and detection of malnutrition risk in participants with low body mass index and eating disorders using 3D optical surface scans. Am J Clin Nutr. (2023) 118:812–21. doi: 10.1016/j.ajcnut.2023.08.004, PMID: 37598747 PMC10797509

[22] Guo Y Zhang M Ye T Wang Z Yao Y. Application of bioelectrical impedance analysis in nutritional Management of Patients with chronic kidney disease. Nutrients. (2023) 15:3941. doi: 10.3390/nu15183941, PMID: 37764725 PMC10537787

[23] Yan KL Liang I Ravellette K Gornbein J Srikanthan P Horwich TB. Body composition risk assessment of all-cause mortality in patients with coronary artery disease completing cardiac rehabilitation. J Am Heart Assoc. (2025) 14:e035006. doi: 10.1161/JAHA.124.035006, PMID: 40008528 PMC12132788

[24] Gordito Soler M Lopez-Gonzalez AA Tarraga Lopez PJ. Association of Sociodemographic Variables and Healthy Habits with body and visceral fat values in Spanish workers. Medicina (Kaunas). (2025) 61:150. doi: 10.3390/medicina61010150, PMID: 39859131 PMC11766553

[25] Alharthy FS Almalki A Alsindi EA Majadah SS Alahmadi SS Alharthy RF. The management of osteoporosis in hospitalized patients with fragility hip fractures in western Saudi Arabia: a real-world tertiary center experience. Arch Osteoporos. (2025) 20:29. doi: 10.1007/s11657-025-01511-w, PMID: 39982553

[26] De Souza MJ Ricker EA Mallinson RJ. Bone mineral density in response to increased energy intake in exercising women with oligomenorrhea/amenorrhea: the REFUEL randomized controlled trial. Am J Clin Nutr. (2022) 115:1457–72. doi: 10.1093/ajcn/nqac044, PMID: 35170727 PMC9170471

[27] Arteaga-Pazmino C Fonseca-Perez D Balladares Mazzini M. Association between dynapenic obesity phenotypes and physical performance in middle-age and older women living in community. Front Nutr. (2024) 11:1480284. doi: 10.3389/fnut.2024.1480284, PMID: 39385775 PMC11461314

[28] Ozkok S Aydin CO Sacar DE Catikkas NM Erdogan T Bozkurt ME. Sarcopenic obesity versus sarcopenia alone with the use of probable sarcopenia definition for sarcopenia: associations with frailty and physical performance. Clin Nutr. (2022) 41:2509–16. doi: 10.1016/j.clnu.2022.09.005, PMID: 36219979

[29] Chondrogianni ME Kyrou I Androutsakos T Panagaki M Koutsompina ML Papadopoulou-Marketou N. Body composition as an index of the trabecular bone score in postmenopausal women. Maturitas. (2025) 198:108273. doi: 10.1016/j.maturitas.2025.108273, PMID: 40286505

[30] Critchlow AJ Alexander SE Hiam DS Ferrucci L Scott D Lamon S. Associations between female sex hormones and skeletal muscle ageing: the Baltimore longitudinal study of aging. J Cachexia Sarcopenia Muscle. (2025) 16:e13786. doi: 10.1002/jcsm.13786, PMID: 40296368 PMC12037696

[31] Chandanwale R Chandanwale K Chandanwale R Chandanwale A. Assessing the correlation between anthropometric measurements and bone densitometry as indicators of bone health in adult women in the community. Cureus. (2024) 16:e68162. doi: 10.7759/cureus.68162, PMID: 39347349 PMC11439113

[32] Brueckheimer PJ Costa Silva T Rodrigues L Zague V Isaia Filho C. The effects of type I collagen hydrolysate supplementation on bones, muscles, and joints: a systematic review. Orthop Rev (Pavia). (2025) 17:129086. doi: 10.52965/001c.129086, PMID: 39980497 PMC11842160

[33] KangMRhoHKimMLeeMLimYChonJ. Effectiveness of protein-enriched oral nutritional supplements on muscle function in middle-aged and elderly women: a randomized controlled trial. J Nutr Health Aging. (2025) 29:100508. doi: 10.1016/j.jnha.2025.100508, PMID: 39951930

[34] Cho S Shin S Lee S Rhee Y Kim HI Hong N. Differential impact of subcutaneous and visceral fat on bone changes after gastrectomy. Endocrinol Metab (Seoul). (2024) 39:632–40. doi: 10.3803/EnM.2024.1956, PMID: 39015029 PMC11375306

